# Medical Society of the State of New York

**Published:** 1903-11

**Authors:** 


### Medical Society of the State of New York.

*Special Meeting, Tuesday Evening, October 13, 1903.*

(*From the Medical Record.*)

THE UNIFICATION OF THE PROFESSION.

A special meeting of the Medical Society of the State of New York was called for the evening of October 13, for the specific purpose of taking steps towards bringing about a union of the two state medical organisations. A good opportunity to secure a large and representative attendance was afforded by the fact that the semiannual scientific meeting of the society was held on the same day.

The large hall of the New York Academy of Medicine was filled with an eager and expectant throng when, at exactly half-past eight o'clock, the gavel fell. If there were any present who feared the audience on this occasion might not be as truly representative as the important business to be transacted demanded, they must have had their fears quickly dispelled on seeing, not only the familiar faces of many eminent members of the local profession, but a goodly proportion of active workers and recognised leaders from distant parts of the State.

On calling the meeting to order, the president, Dr. A. T. Bristow, of Brooklyn, said:

*Gentlemen of the Medical Society of the State of New York:*

Almost two years ago the Medical Society of the State of New York, acting upon the recommendation of the president, Dr. Henry L. Elsner, authorised the appointment of a committee for the purpose of promoting a union between the state society and the state association. On request of our society, the association appointed a similar committee. Meetings of two committees were held from time to time until, in October, 1902, at this annual meeting, the association adopted resolutions placing restrictions upon its committee from which it could not deviate. At this time, also, the uncertain condition of the Code of the American Medical Association rendered the position of our committee difficult, for no one seemed to know its exact status. The clause, which was so objectionable to the state society, remained unchanged. An additional obstacle was the request of the association that the two societies apply for a new charter. In the course of time, under the resolutions referred to, the committee of the association went out of existence.

At the May meeting of the American Medical Association the clause of the old code, which had caused the division of many years ago, was stricken out entirely, and it was clearly stated that the new "principles of ethics" were merely advisory, and not obligatory on state bodies.

Your conference committee, recognising the fact that a most serious obstacle to the wish for union had been swept aside, requested the association to name a new committee to the end that fresh efforts might be made to close the gap. Informal conferences developed the fact that a reasonable compromise could be obtained by which the wish of the association could be complied with, and that some legal recognition be taken of the new order of things without in any respect sacrificing the continuity of our charter.

At a special meeting of the Council and Fellows of the association, the resolutions were unanimously passed which were printed in the notice of this special meeting (*Medical Record,* October 10, page 581), and a new committee was appointed.

I ask you to notice that these resolutions contain but a single restriction, *i. e.,* that the reorganised society shall be called The Medical Society of the State of New York. (Applause). In all other respects the new committee of the association is absolutely untrammeled, and has been appointed with power.

The request is made that your present committee be given similar power. This is the question which you are called upon to decide tonight. The association has reposed full trust and confidence in its committee for the purpose of furthering that union which, two years ago, The Medical Society of the State of New York expressed its anxiety to bring about.

It now remains for the society, which made the original proposition looking toward peace, to say whether it will have peace and bring to a happy termination a conflict which has lasted almost a quarter of a century.

I will ask Dr. Curtis to read the communications of the association.

The secretary then read the resolutions which have already been printed (*Medical Record, Ibid.*). They were received with hearty applause.

Dr. D. B. St. John Roosa then said:

*Mr. President, and Fellow-Members:*

As one of those who has lived long enough to see the beginning, and I hope will live long enough to see the ending of this controversy that has been carried on so long, I have the pleasure of offering the following resolution, which, I think, will have the vote of every member of The Medical Society of the State of New York:

*Whereas,* The New York State Medical Association, at a recent special meeting, duly assembled, has, by unanimous vote, appointed a committee with full power to meet with the similar committee of the Medical Society of the State of New York to arrange for the unification of the two organisations under the corporate name of The Medical Society of the State of New York; therefore, be it

*Resolved,* That the committee of conference of the Medical Society of the State of New York, already appointed, be given power equal to, and commensurate with the powers recently granted the committee created by the New York State Medical Association for the purpose of unifying the two State Medical Societies into the Medical Society of the State of New York.

Dr. Willis G. Macdonald, of Albany, said:

*Mr. President and Gentlemen of the Medical Society of the State of New York:*

I rise in my place to heartily second the preamble and resolution presented by that distinguished fellow of this society, Dr. Roosa. (Applause.) The Medical Society of the State of New York has seen many glorious occasions. There are numerous notable instances in its long history of value to the American medical profession—its notable relation to the very organisation of the American Medical Association; its later relations in the discussion of the very subject matter which has separated us in the State of New York for so many years; its more intimate relations with the medical education and the adoption of a method in this state which has been the pattern of many others. Again, and perhaps the most notable occasion—the best work of this society—presents itself now on this occasion. It shows you, Mr. President, that the medical profession, besides being learned, is also great; it shows you, Mr. President, that physicians can forget their differences; and that we are no longer the by-word of the world; it shows you, Mr. President, that now we are able to agree and sit down together in unity; it shows you that with the carrying of this motion men are able to forget and to forgive; it shows you that it is possible for us at this time to so meet together that we can settle all of our differences upon a very easy and very beautiful plan. (Applause.)

Gentlemen, this is the most delightful occasion which I have ever known in the history of the Medical Society of the State of New York, and, as I have already said to you, I leave it to you if it is not the most important in its entire history.

Gentlemen, we stand at the beginning of the twentieth century,—on the very threshold of it,—with all the progress that has gone before, with all the attainment in a science of the immediate past, only to look forward as the unified profession to our greater opportunities, and to the greater necessities and to the greater honors of the future.

Mr. President, it is my pleasure to again second this resolution.

The resolution was read again by the president. It was adopted, without a dissenting vote, amid cheers and prolonged applause, and the adjournment of the meeting was followed by hand-shakings and general congratulations. Whatever may be the final outcome, no one who witnessed the unmistakable evidences of rejoicing and good fellowship exhibited after the adjournment of this memorable meeting could fail to be impressed with the fact that many of the old contentions had become dead issues, and that the rank and file of the profession, at least, were weary of bickerings and petty politics, and anxiously awaited the time when they would be relieved from this thraldom and could follow untrammeled those lines which were more in keeping with the seriousness and dignity of their noble calling.

